# In Vitro MRS of Cells Treated with Trastuzumab at 1.5 Tesla

**DOI:** 10.3390/ijms25031719

**Published:** 2024-01-31

**Authors:** Wiesław Guz, Rafal Podgórski, Zuzanna Bober, David Aebisher, Adrian Truszkiewicz, Marcin Olek, Agnieszka Machorowska Pieniążek, Aleksandra Kawczyk-Krupka, Dorota Bartusik-Aebisher

**Affiliations:** 1 Department of Diagnostic Imaging and Nuclear Medicine, Medical College of Rzeszów University, 35-959 Rzeszów, Poland; wguz@ur.edu.pl; 2 Department of Biochemistry and General Chemistry, Medical College of Rzeszów University, 35-959 Rzeszów, Poland; rpodgorski@ur.edu.pl (R.P.); dbartusikaebisher@ur.edu.pl (D.B.-A.); 3 Department of Photomedicine and Physical Chemistry, Medical College of Rzeszów University, 35-959 Rzeszów, Poland; zbober@ur.edu.pl (Z.B.); atruszkiewicz@ur.edu.pl (A.T.); 4 Department of Densitry, Faculty of Medical Sciences in Zabrze, Medical University of Silesia, 40-055 Katowice, Polandagamach@onet.pl (A.M.P.); 5 Department of Internal Medicine, Angiology and Physical Medicine, Center for Laser Diagnostics and Therapy, Faculty of Medical Sciences in Zabrze, Medical University of Silesia, 40-055 Katowice, Poland

**Keywords:** MRS, Trastuzumab, breast cancer

## Abstract

The aim of the study was to investigate the effect of Trastuzumab on the MCF-7 and CRL-2314 breast cancer cell lines. Additionally, an attempt was made to optimize magnetic resonance spectroscopy (MRS) for cell culture studies, with particular emphasis on the impact of treatment with Trastuzumab. The research materials included MCF-7 and CRL-2314 breast cancer cell lines. The study examined the response of these cell lines to treatment with Trastuzumab. The clinical magnetic resonance imaging (MRI) system, OPTIMA MR360 manufactured by GEMS, with a magnetic field induction of 1.5 T, was used. Due to the nature of the tested objects, their size and shape, it was necessary to design and manufacture additional receiving coils. They were used to image the tested cell cultures and record the spectroscopic signal. The spectra obtained by MRS were confirmed by NMR using a 300 MHz NMR Fourier 300 with the TopSpin 3.1 system from Bruker. The designed receiving coils allowed for conducting experiments with the cell lines in a satisfactory manner. These tests would not be possible using factory-delivered coils due to their parameters and the size of the test objects, whose volume did not exceed 1 mL. MRS studies revealed an increase in the metabolite at 1.9 ppm, which indicates the induction of histone acetylation. Changes in histone acetylation play a very important role in both cell development and differentiation processes. The use of Trastuzumab therapy in breast cancer cells increases the levels of acetylated histones. MRS studies and spectra obtained from the 300 MHz NMR system are consistent with the specificity inherent in both systems.

## 1. Introduction

Magnetic resonance imaging (MRI) is an essential tool in the arsenal of both physicians and scientists in the natural sciences. It provides invaluable services in imaging both the structures of the human body and in showing changes occurring at the cellular level. The acquired spectroscopic spectra make it possible to track chemical changes, analyze the composition of the tested objects or changes occurring in cells in response to pharmacological treatment. Magnetic resonance spectroscopy (MRS) was used in this study to illustrate the effect of the drug Trastuzumab on breast cancer cells.

**Figure 1:**
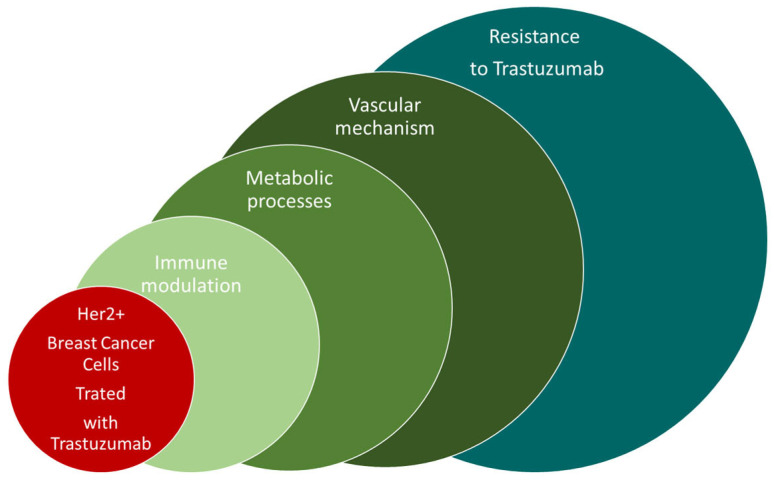
The presentation of factors with influence on the development of Trastuzumab resistance.

Breast cancer is the most common type of cancer among women [1,2,3], affecting over two million women each year. At the beginning of 2021, according to data from WHO, breast cancer is the most frequently diagnosed cancer in the world. It is diagnosed every year in approximately 12% of women worldwide. Early diagnosis [4] and the implementation of an appropriate treatment regimen as well as therapy monitoring [5] are important. We must pay attention to the importance of HER receptors in the pathogenesis of breast cancer. The HER receptor family includes the following receptors: HER1, HER2, HER3, HER4. Too many copies of the ERBB2 gene encoding the HER2 protein result in its overexpression in cancer cells [6,7,8,9]. Overexpression of the HER2 receptor is found in 15% of breast cancer cases. Overexpression of the HER2 receptor influences the course and treatment of breast cancer, results in a worse prognosis, and significantly influences the aggressiveness of the cancer. Signaling pathways also influence the effectiveness of treatment. Herceptin-based therapies are part of many treatment regimens. Immunotherapies used in treatment are most often based on monoclonal antibodies, such as Trastuzumab of the IgG1 class, which has a therapeutic effect against the positive domain of the HER2 receptor [10]. Due to the fact that breast cancer includes several subtypes, they are characterized by different receptor expression. Breast cancer with HER2 overexpression is an increasingly common type of cancer, therefore HER2-targeted therapy is necessary in the commonly used treatment of this type of cancer. The implementation of tests using a generally available and safe diagnostic method, i.e., MRS, will allow for the monitoring of the therapy and assessing its effectiveness. In this paper, we present spectroscopic studies of the most frequently used drug, Trastuzumab, and studies using this drug in therapy in breast cancer cell lines. Trastuzumab is an IgG1 antibody that has therapeutic activity against the positive domain of the HER2 receptor. Currently, it is believed that the biological drug Trastuzumab is effective only in patients whose cancer overexpresses the HER2 receptor. This humanized antibody against the extracellular domain of the HER2 receptor binds to the membrane-bound subdomain IV of the extracellular portion of the HER2 protein. By connecting to the receptor, it inhibits the transmission of division signals to the cell nucleus, thereby slowing down the development of cancer. When HER2 is overexpressed, cancer cells multiply at a higher rate due to the higher growth factor. Moreover, they show greater drug resistance. The main indications for the implementation of treatment with Trastuzumab are the treatment of breast cancer with overexpression of the HER2 receptor, palliative treatment, early stage of cancer, breast cancer with metastases, and gastric cancer with metastases. The monoclonal antibody Trastuzumab is a mirror image of the protein receptor that can attach to the receptor and thus block the growth factor, slowing down the development of the tumor. In the case of overexpression, there is a chance of a positive response to therapy with Trastuzumab. The use of Trastuzumab improved the results of breast cancer treatment. However, a large proportion of patients after Trastuzumab treatment develop primary resistance [11]. Figure 1. The presentation of factors with influence on developing of Trastuzumab resistance. Despite improved results in breast cancer treatment with Trastuzumab, most patients with metastases experience disease progression due to primary or acquired resistance. In Trastuzumab-treated patients with locally advanced or metastatic HER2+ breast cancer, an objective response rate (ORR) of only 65% was observed [12].

Nuclear magnetic resonance spectroscopy (MRS) is a technique used mainly in clinical trials to study brain tumors, metabolic changes in cancer, but also multiple sclerosis, stroke, Alzheimer’s disease, and others. Thanks to this method, we can obtain information about the biochemical composition of the examined area by assessing the concentration of selected metabolites. MRS allows obtaining information about the biochemical composition by assessing the concentration of selected metabolites. The introduction of pulsed MR spectroscopy sequences into clinical trials makes it possible to obtain a spectrum of the brain. MRS, unlike MR imaging, shows the distribution of metabolites, not only morphological and functional information. The obtained spectrum carries information about the concentration of metabolites, the range of which is expressed in ppm (parts per million), and carries information about the chemical shift. The use of the MRS technique to study cell cultures and the assessment of their metabolism after therapy provides the potential to transfer research from in vitro to in vivo. The aim of the study was MRS research using a 1.5 T magnetic resonance imaging device in cell cultures and the examination of their metabolites before and after Trastuzumab treatment.

## 2. Results

**Figure 2:**
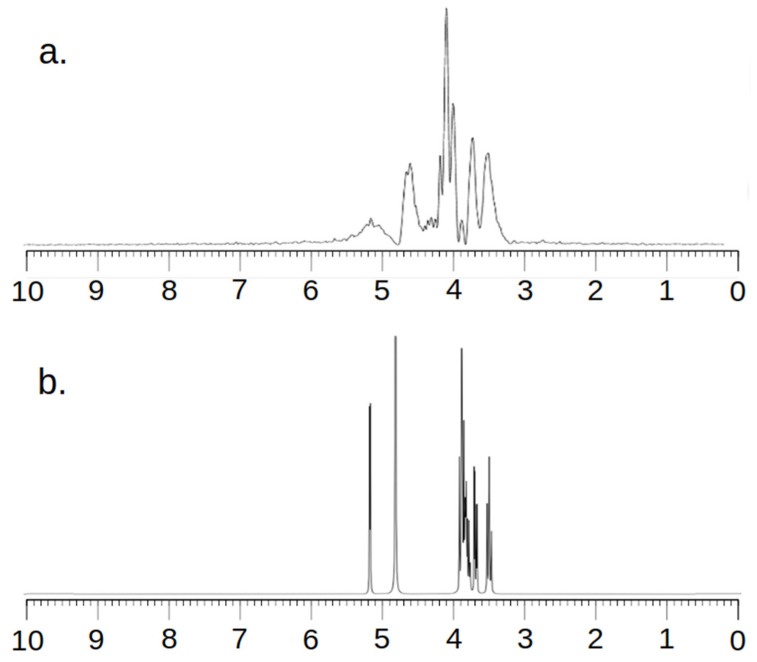
MR spectra (**a**) and NMR spectra (**b**) of Trastuzumab.

**Figure 3:**
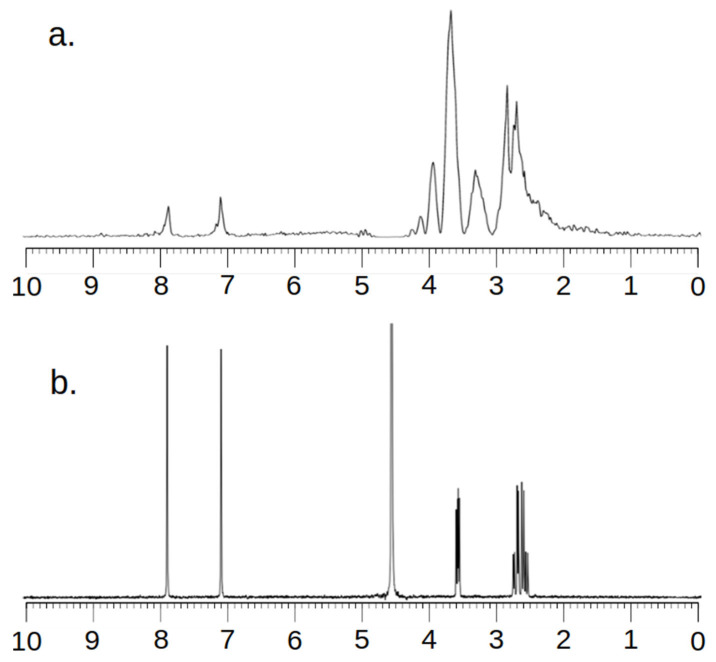
MR Spectra (**a**) and NMR Spectra (**b**) of L-Histidine.

Taking into account the fact that an MRS examination is a relatively low-resolution examination, it was necessary to ensure the correctness of the research method. For this purpose, spectra were recorded in the MRS and NMR systems. Figure 2 and Figure 3 below show the obtained spectra.

All MRS examinations were performed using the SV sequence and a constructed single-channel coil for examining small objects. First, MRS tests were performed on a sample containing 1 mg of Trastuzumab dissolved in 1 mL of clean water (Figure 2).

Analyzing the images, one can see a high agreement between the spectra obtained by the MRS and NMR methods. The lack of a signal in the vicinity of 5.2 ppm and 4.8 ppm for Figure 2 and 4.8 ppm for Figure 3 needs to be explained. To obtain spectra using the MRS method, water attenuation must be turned on. This is performed in both the MR system and the SAGE 7.7.1 software used for analysis. This software eliminates water using a digital method. A characteristic feature of such filtering is the fact that the water peak area is strongly suppressed. The signal of both water and all spectrum components located near the water point is attenuated. The above statements correlate perfectly with the obtained spectra and are consistent with the specificity of both systems.

**Figure 4:**
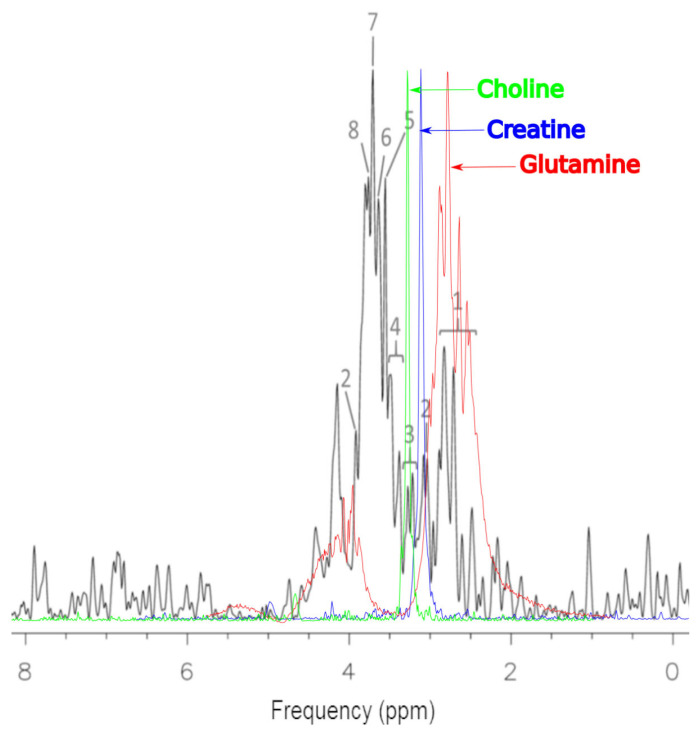
Representative ^1^H MRS of cells MCF-7 samples before treatment. The cells were properly cleaned by washing with PBS. There was no residual drug or culture medium left before preparing them for spectroscopy: 1—glutamine; 2—creatine; 3—total choline; 4—taurine; 5—glycine; 6—PCho; 7—GPC; 8—alanine.

The MRS of cancer cells offered rapid access to the metabolic content in cell samples before and after treatment with Trastuzumab. Single-voxel MRS with an engineered single-channel coil (Figure 4) showed the ability to detect cellular composition in small samples with a 1.5 T MRI scanner. MRS tests were performed for the drug components: D-Trehalose dihydrate > 99%, L-histidine > 99% (TLC) (Figure 4), Polysorbate 20. Optimal concentrations of 1 mg/1 mL of pure water were prepared.

When performing the analysis, you can see a very low level of the signal coming from the water thanks to the automatic use of digital signal filtering.

Then, the same concentrations were prepared in D_2_O and experiments were performed using a 300 MHz NMR from Bruker (Billerica, MA, USA). This spectrum was used as a standard for the spectrum obtained by MRS. The use of pure water instead of D_2_O in the MRS method was dictated by the fact that the cells were cultured in H_2_O and there was a need to obtain such a spectrum.

### 2.1. MCF-7 Cells

The representative ^1^H MRS of MCF-7 cells before treatment is presented in Figure 4 The processed spectra were prepared for metabolite identification and analysis in untreated MCF-7 cells. For the identification of basic metabolites in the MRS of MCF-7 cells, the Bruker Human Metabolome Database (HMDB) metabolite library was used to match the spectra. The contributions of the metabolites were found to be more significant than the contribution of the other expected molecular cancer markers. In untreated MCF-7 cells, we detected: glutamine, creatine, total choline, taurine, glycine, PCho, GPC, and alanine. MRS studies are extremely useful in the detection of similar groups of glutamine, creatine, total choline, taurine, glycine, PCho, GPC and alanine in vitro. Herein, there is a known problem with the numbers of overlapping peaks. In this situation, the identification of large numbers of metabolites can be difficult. MRS is acquired as one dimensional (1D) spectroscopy. However, two dimensional (2D) spectroscopy studies can help to overcome these problems. The use of 2D MRS in metabolomics is usually restricted to the characterization of unidentified compounds from the 1D spectra.

Shown in Figure 4, the colored spectra are images of elements superimposed on the basic spectrum, which represents the MCF-7 culture before treatment (black). These spectra were obtained as a result of work on this project and the scanning of choline, creatinine and glutamine solutions. The reason for such actions was the need to visualize these compounds in the system used to conduct the research. The 1.5 T system has a relatively low resolution for imaging such complex solutions. The peak heights of the spectra have been intentionally increased due to the need to show them more precisely against the background of the basic spectrum.

**Figure 5:**
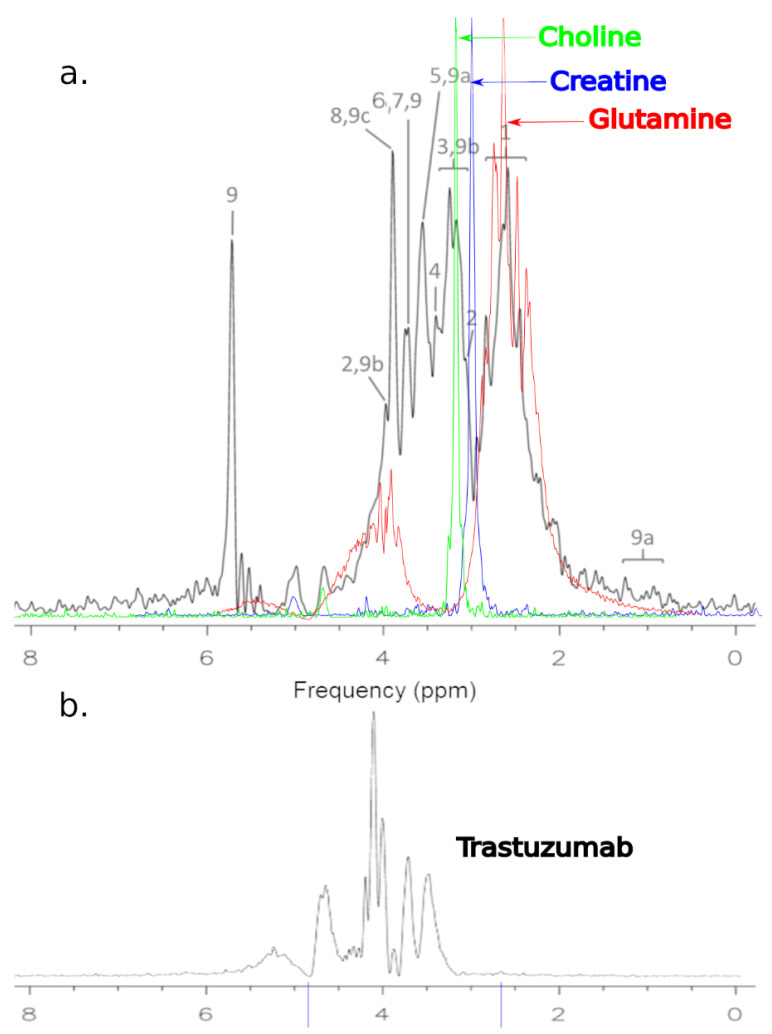
(**a**) Representative ^1^H MRS of cells MCF-7 after treatment: 1—glutamine; 2—creatine; 3—total choline; 4—taurine; 5—glycine; 6—PCho; 7—GPC; 8—alanine; 9—Trastuzumab; 9a—pylosorbate 20; 9b—L-Histidine; 9c—D-Trehalose. (**b**) Spectrum of Trastuzumab.

Changes in the spectrum were noticed after Trastuzumab treatment (Figure 5). Our analysis of the MCF-7 cells after treatment with Trastuzumab was found to be different from the untreated cells. Moreover, ten metabolites showed unique changes in abundance when treated with Trastuzumab.

In Figure 5a, the spectroscopic spectrum is presented, which constitutes the superimposed elements of the spectra of choline, creatine, and glutamine on the basic spectrum, which represents the MCF-7 culture after treatment (black). In Figure 5b, the spectrum of Trastuzumab obtained as a result of MRS scanning in a 1.5 T MR system is shown. You can see that the drug peaks stand out in the cell culture signal. The Trastuzumab signal is lower due to its concentration.

Table 1 shows a summary of the detected metabolites before and after treatment with Trastuzumab. We used MRS as the first screening for MCF-7 cells before and after treatment. Sample preparation was relatively simple when compared to other analytical methods. However, metabolite screening requires maximum sensitivity with a broad compound coverage, thus we built a suitable coil and optimized the parameters of the MRS measurements.

### 2.2. CRL-2314 Cells

Based on the results regarding their number, it can be concluded that the treatment of CRL-2314 cells with the drug Trastuzumab (0.024 mg/mL) did not have the expected effect. Moreover, we can observe that total cell number did not change or increased. It may be related to the fact that Trastuzumab binds to the HER2 receptor, and CRL-2314 cells express this receptor poorly, hence the negligible effect of the therapy. 

After a 2-fold Trastuzumab dose increase and 24 h of incubation, a significant increase in viable cells and total cells was noted. A dose that was successively doubled up to 0.048 mg/mL resulted in a decrease in viable cells and total number of cells. The existing effect proves the inhibition of cell growth and a reduction in their number, and thus confirms the therapeutic effect (Table 2).

The dosage of Trastuzumab is coupled to the efficiency of treatment as well as to changes in metabolites. Trastuzumab treatment shows perturbations in mitosis and necrosis with low doses, and increased levels of cell cycle arrest and apoptosis as the dose increases.

**Figure 6:**
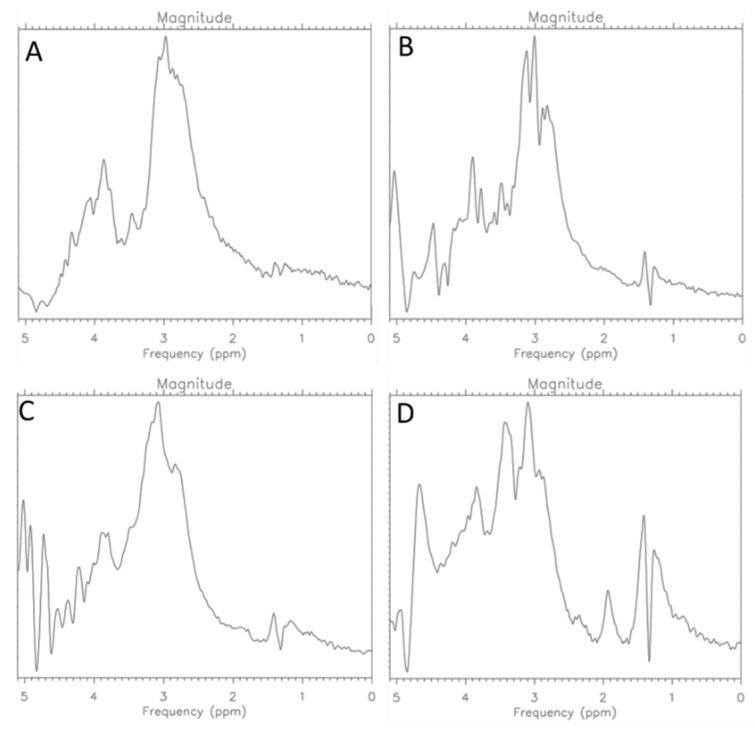
MR Spectra for CRL-2314 (**A**) cells, CRL-2314 cells + 0.012 mg/mL Trastuzumab (**B**), CRL-2314 cells + 0.024 mg/mL Trastuzumab, (**C**) and CRL-2314 cells + 0.048 mg/mL Trastuzumab (**D**).

Spectroscopic examinations were performed simultaneously. The spectra obtained are presented in the figure below (Figure 6).

On the basis of the presented spectra (Figure 3), an increase in the signal was observed at 1.9 ppm, which proves the induction of histone acetylation. In scientific publications, we can find confirmation that SNHG14 lncRNA promotes breast cancer and Trastuzumab resistance by regulating PABPC1 expression through H3K27 acetylation [13]. Moreover, the applied therapies may increase acetylation and disturb the stability of HER2 [13].

**Figure 7:**
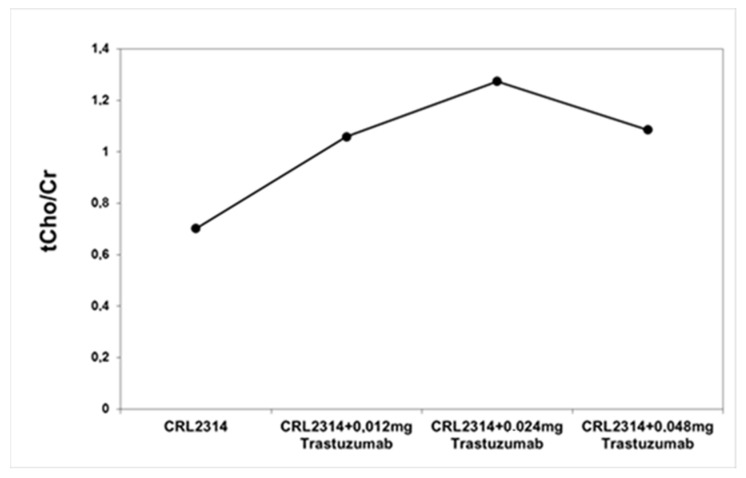
The ratio of choline to creatinine for breast cells CRL-2314 before and after treatment with the drug Trastuzumab dissolved in the culture medium.

The analysis of the obtained spectra allowed us to determine the ratio of choline and creatinine. Based on the obtained results, the ratio of choline to creatinine was determined for CRL-2314 breast cancer cells before and after treatment with Trastuzumab dissolved in the culture medium. The obtained results are shown in the figure below (Figure 7).

In the second step, a solution of Trastuzumab in an injection was prepared, results regarding the number of cells are presented in Table 3.

**Figure 8:**
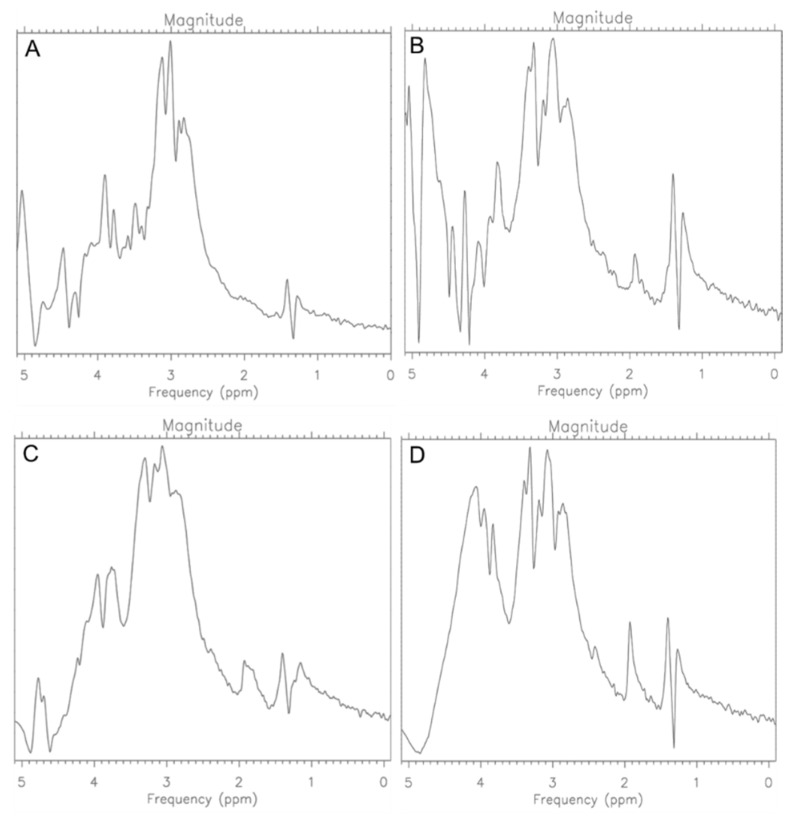
MR Spectra for CRL-2314 (**A**) cells, CRL-2314 cells + 0.012 mg/mL Trastuzumab (**B**), CRL-2314 cells + 0.024 mg/mL Trastuzumab, (**C**) and CRL-2314 cells + 0.048 mg/mL Trastuzumab (**D**). The drug is dissolved in a liquid for injection.

The same doses of Trastuzumab were used as in the previous step, but they prepared in liquid for injection unlike in step 1. Based on the abundance measurements, a slight increase in viable cells and total cells was noticed after the 0.12 mg/mL dose, which indicates the activation of cell growth. Then, after applying a double dose and 24 h of incubation, another increase in the number of cells was noticed, which confirms the previous step of using too low a dose. However, after using the drug dose of 0.048 mg, an evident therapeutic effect was noticed. This is also confirmed by the spectroscopic spectra (Figure 8).

**Figure 9:**
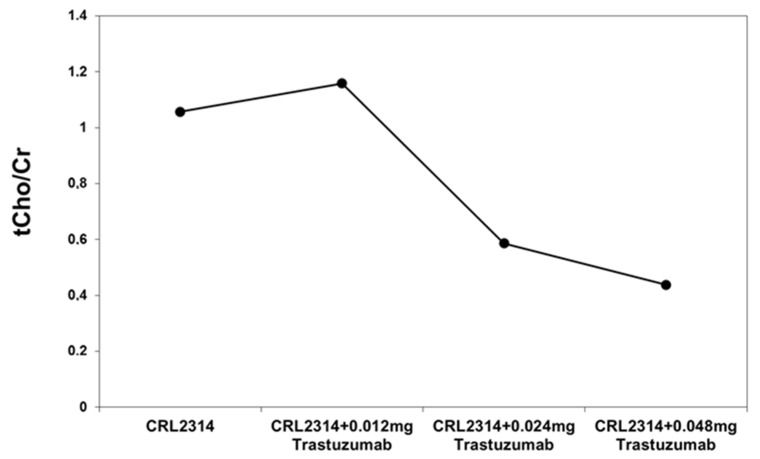
The ratio of choline to creatinine for CRL-2314 cells before and after treatment with Trastuzumab dissolved in injection fluid.

The analysis of the obtained spectra allowed us to determine the ratio of choline and creatinine. The obtained results are shown in the figure below (Figure 9).

Our results indicate that Trastuzumab treatments had an impact on the metabolic profile of breast cancer cells. However, there were differences between MCF-7 and CRL-2314 cells. The differences arise from the fact that the cell lines exhibit pathological and clinical features, as they possess different hormonal statuses and receptor types for drug targets [14,15]. MCF-7 cells are known to express high levels of estrogen receptors and are reliant on estrogens for their growth. In contrast, SkBr3 cells are estrogen-independent and estrogen receptors are not expressed [16].

## 3. Discussion

The MRS of breast cancer has received increased interest by clinicians and researchers due to the application of MRI to breast cancer. Many hardware and software advances are now taking place that are improving the quality and reliability of breast MRI and MRS. For example, we noticed significant improvements in the signal-to-noise ratio that can be achieved by using multi-channel phased-array coils that are now commercially available. MRI is a powerful noninvasive clinical technique for the analysis of cancer cell receptor targeting in vivo and has demonstrated potential for aiding in improvements in immunotherapy. Using the MRS method, cell destruction tests were performed, and in the future, there is a possibility to switch these tests into in vivo tests. Due to the fact that in recent years, we have observed an increasing number of cases of breast cancer, research may contribute to a better understanding of the drug and the optimization of therapy. This suggests that this project will provide solutions for filling the gap between Trastuzumab efficiency and Trastuzumab delivery control. We chose the conventional drug Trastuzumab for this study to pinpoint its potential to be a more effective drug for breast cancer patients.

Various drugs have been studied for the treatment of the Her-2 receptor; however, Trastuzumab was the first immunotherapeutic treatment targeting the HER-2 receptor [17,18]. Although Trastuzumab is an efficient drug, resistance to Trastuzumab is a common and challenging problem. Data show that Trastuzumab reduces breast cancer recurrence by ~50% [19,20].

MRS helps assess the metabolic status of malignant, benign, and normal breast tissues. In vivo breast MRS shows high levels of choline-containing metabolites (tCho), indicating the rapid proliferation of malignant tumors [20,21]. In that research paper, the group found elevated glycine in the patient based on in vivo MRS. High glycine levels may imply a worse prognosis [22].

Amino acids play a key role in the growth of cancer cells. Many studies show changes in the levels of metabolites in the breast tissues of patients with breast cancer [23]. Some of the most important amino acids are glucose and glutamine, which enable cell proliferation and survival [24]. In addition, glutamine allows for the import of essential amino acids, which in turn prevents apoptosis in cancer cells [25]. In cell cultures carried out under in vitro laboratory conditions, glutamine is supplied with the culture medium. On the other hand, in neoplastic tissues in vivo, a much lower level was observed as compared to the surrounding tissues, which may indicate a greater demand by neoplastic tissue [26]. In addition to the importance of the amino acid glutamine in the growth and survival of cancer cells, a decrease in blood glutamine levels has been observed in cancer patients, which may indicate tumor progression. Cell changes in amino acids may indicate a disturbance in cell proliferation or cell death. The metabolic changes in cancer cells are the result of oncogenic changes that affect the cell signaling pathways that lead to cell growth and proliferation. Increased uptake of amino acids, for example glutamine, can be observed in many types of cancer [27]. Benzylserine treatment was shown to significantly inhibit the uptake of leucine and glutamine in MCF-7, HCC1806, and MDA-MB-231 breast cancer cells by disrupting amino acid homeostasis, leading to cell death [28]. In contrast, HER2 breast cancer with CDK12 enhancement is resistant to HER2+ targeted therapy [29]. In other studies, NMR spectra showed that phosphocholine signals were stronger in control samples than in cells after tamoxifen/cisplatin therapy, after which they decreased significantly [30]. One of the methods used to assess metabolic changes is MRS. Euceda et al. assessed metabolic changes during neoadjuvant chemotherapy combined with bevacizumab. Increased levels of phosphocholine (PCh), glycerophosphocholine (GPC), and taurine (Tau) were noted in TP1, while the levels of glucose (Glc) and lipids were higher with increasing treatment duration [31]. According to the Warburg effect, neoplastic cells metabolize glucose to lactate [32]. In their work, Bathen et al. demonstrated the possibility of using HR MAS MRS for the differentiation of breast tumor and healthy tissue on the basis of metabolic profiling [33]. By contrast, Cheng et al. presented the use of MRS for the assessment of breast cancer with metabolites in the samples. Attention was paid to phosphocholine, lactate, and lipids, and a correlation between tumor metabolism and the degree of malignancy was noted. Phosphocholine is regarded as a biomarker of the malignant transformation of breast cancer [34]. Also, Sitter et al. noticed higher choline and glycine concentrations in tumors larger than 2 cm compared with smaller tumors based on HR MAS MR spectroscopic studies [35]. Choi et al. used the HR-MAS MR method to study breast cancer samples taken from core biopsy, and higher concentrations of Cho, Cr, and Tau were found in PR-negative tumors than in PR-positive tumors [36]. Moreover, Sitter et al. demonstrated lower glycine concentrations in patients with a good prognosis (1.1 µmol/g) compared to patients with a poor prognosis (1.9 µmol/g) [37]. In addition, higher levels of glycine and lactate could be associated with lower survival rates suggesting that the metabolic status of the tumor may be helpful in determining patient prognosis [38]. The Demas group showed an increase in glutamate after the administration of 500 nM CB-839 for 72 h; in this study, the treatment significantly increased glutamine levels in breast cancer cells [39]. However, in other studies, increased glucose consumption and lactate excretion were noted after 48 h therapy of MDA-MB-231 or MCF-10A breast cancer cells with betulinic acid (BA) [40]. In studies, we can also find reports of the monitoring of the concentration of extracellular metabolites collected with microdialysis probes from rat brains. Based on the ^1^H MRS studies, higher concentrations of lactate and meglumine were found in an animal model of glioma after anticancer drug administration compared to healthy tissue [41].

The study also evaluated the potential of 1 H NMR to assess plasma metabolism. Studies have been performed assessing disease progression by evaluating the metabolome of early and late-stage breast cancer [42,43,44]. Suman et al., based on the NMR spectrum, noted changes in more than a dozen metabolites that show differences between early stage and late-stage breast cancer. They noted an increase in lactate in the early BC group compared to the control group, and an even greater increase for late BC. Late BC was characterized by high glucose levels compared to the early BC group and the control group. Changes were also observed for glycine, lysine, valine, and other metabolites [45]. 

Similarly, a comparison of the metabolome of filtered plasma from EBC and MBC patients showed that Lac showed an inverse correlation with tumor size in EBC [46].

Summing up, the observed increase in metabolites with the progress of treatment with Trastuzumab in breast cancer cell culture is characteristic of cancer cells. The glycolytic phenotype is designed to meet high energy requirements. On the other hand, the increase in glutamine levels with the use of Trastuzumab treatment indicates a decrease in glutaminolysis. Glutamine is involved in the biosynthesis of nucleotides, lipids, and proteins [47]. On the basis of the performed studies, changes in amino acid metabolism were identified as a possible effect of Trastuzumab therapy in breast cancer cell cultures overexpressing the HER2 receptor.

The challenge was to determine the optimal growth conditions and viability of the breast cancer cell culture for MRS measurements. The next challenge was to determine the optimum concentrations of Trastuzumab–dendrimer–fluorine for treatment in the 3D HBR device. Previous studies of breast carcinoma report that Trastuzumab alone did not inhibit growth by more than 30–50% in relation to doses of 10 µg/mL–1000 µg/mL over 72 h [48]. By using Trastuzumab at concentrations of 0.05 µg/mL–1000 µg/L over 72 h, we obtained MCF-7 cell growth inhibition of 26 ± 3% to 46 ± 5% [49].

In clinical trials, biomarkers allow for better assessment of the therapeutic response using diffusion-weighted (DW)-MRI apparent diffusion coefficient (ADC) studies, as well as fluorodexoyglucose (FDG), fluorothymidine (FLT), and fluoromisonidazole (FMISO)-PET imaging [50]. Numerous studies show the use of ^18^F-FDG-PET for the monitoring of cancer cell response to targeted therapies in the Wilms mouse tumor model [51]. In other studies, we can find a comparison of GlucoCEST MRI and ^18^F-FDG-PET to assess the response to chemotherapy and metabolic therapies in breast cancer studies in mice, with the studies confirming that glucoCEST can be a sensitive technique for monitoring metabolic pathways [52]. Also, Kristian et al. presented the use of dynamic ^18^F-FDG-PET to study glucose distribution in tissues in order to monitor the effects of therapy. Based on the research, it was found that dynamic PET can detect changes in tumor perfusion and metabolism after anti-angiogenic therapy in murine models [53]. In other studies, researchers demonstrated the use of granzyme B-specific (GZP-PET) imaging to monitor changes in effector cell activation in response to treatment with paclitaxel (PTX) and immunological inhibitors in a mouse model of triple-negative breast cancer [54]. Other studies used integrative high-performance liquid chromatography in conjunction with quadrupole tandem mass spectrometry (HPLC-QTOF) to assess metabolic changes in MCF-7 cells after R-metalaxyl and S-metalaxyl therapy [55]. The study of metabolites after treatment with Trastuzumab is also performed using ultra-high liquid time-of-flight mass spectrometry (UPLC-TOF-MS) [56]. NMR based studies have shown that the selection of the culture medium turns out to be important and the concentration of many metabolites will largely affect the metabolic content [57], and the same was confirmed by liquid chromatography studies [58]. In addition, it has been shown that, depending on the type of culture, the activity of the drug T-DM1 is different in 3D culture, most likely due to tumor heterogeneity, which may contribute to progress in future studies on therapeutic effects and resistance to therapy [59]. In terms of resistance testing, mucin 1 (MUC1) is a potential target for overcoming Trastuzumab resistance in breast cancer therapy [60]. The study investigated the changes in metabolism induced by the BPTES inhibitor in two human breast cancer cell lines, MCF-7 and MDA-MB231, and the non-cancerous line MCF-10A. NMR spectroscopy was performed in conjunction with the labeling of isotopes. Metabolism changes induced by BPTES were found in tumor cell lines, especially under hypoxic conditions, and in addition, about 30% of metabolites changed compared to the non-tumor line [61]. Sharma, on the other hand, described the use of MRS to determine the metabolism of breast cancer [62].

After drug treatment, cancer cells undergo reprogramming, which changes metabolic flows. Increased glycolysis, increased production of lactic acid, restructuring of the Krebs cycle, and decreased availability of oxygen are observed in the cells. Lactic acid is more toxic to adjacent normal cells than to a malignant tumor that has developed survival mechanisms. In addition to lactic acid, CO_2_ is a significant source of acidic extracellular pH (pHe) in the tumor microenvironment; in the reaction catalyzed by carbonic anhydrase (CA), CO_2_ is hydrated, resulting in the formation of bicarbonate (HCO^−3^) and H+. Cancer cells undergo metabolic reprogramming to modify metabolic fluxes in response to high demands not only for ATP, but also NADPH, NADH, and carbon skeletons [63].

Acetylation neutralizes the positive charge of lysine thereby causing histones to drift away from DNA, which has a negative charge. Changes in histone acetylation play a very important role in both the development and differentiation of breast cancer cells. It has been proven that the use of Trastuzumab therapy in breast cancer cells increases the levels of acetylated histones. Scientific publications confirm that lncRNA SNHG14 promotes breast cancer tumorigenesis and Trastuzumab resistance by regulating PABPC1 expression through H3K27 acetylation [64]. Moreover, the therapies used may increase acetylation and disturb the stability of HER2 [65]. MRS in vitro showed the possibility of obtaining information about the chemical content of breast cancer cells using a 1.5 T clinical apparatus. This information can be used further for clinical applications, such as monitoring the response to cancer therapies and improving the accuracy of lesion diagnosis. Initial MRS studies of breast cancer show promising results, and a growing number of research groups are incorporating the technique into their breast MRI protocols.

MRS is a key method in metabolomics and the technology continues to improve allowing NMR spectroscopy to remain involved in future drug research and development and facilitating the movement toward personalized medicine.

## 4. Materials and Methods

The MCF-7 cell line (American Type Culture Collection, Manassas, VA, USA) was purchased from Sigma Aldrich (St. Louis, MO, USA). For the cultivation of this cell line, EMEM medium (EBSS) + 2 mM. Glutamine + 1% NEAA essential amino acids + 10% FBS fetal bovine serum were used. The CRL-2314 line and breeding media were obtained from ATCC (American Type Culture Collection (ATCC^®^)), P.O. BOX 1549, Manassas, VA 20108, USA, were purchased through LGC standards (Łomianki, Poland). Sodium bicarbonate was obtained from Honeywell Fluka, Collagen bovine type Lyophilized Fibrous Powder from Tendon was provided by Advanced BioMatrix (St, Louis, MO, USA), while Penicillin-Streptomycin-Neomycin Solution Stabilized, Fetal Bovine Serum (FBS), and Epidermal growth factor (EGF) were provided by Sigma Aldrich and were used to prepare the complete growth media for the cells under sterile conditions in an Alpina laminar chamber. Both cell lines were grown under standard conditions: 37 °C, 5% CO_2_ and 95% humidity.

Human Gland Mammary Adenocarcinoma Cells (MCF-7), is a Her-2 producing cell line and was commercially available. The Her-2 overexpression of these cell lines was confirmed using cytometric analysis. Carbon dioxide-independent medium supplemented with 10% buffered fetal bovine serum (FBS), 1% L-Glutamate, and 1% antibiotic (penicillin/streptomycin) was used. The cells were suspended in a CO_2_ independent medium and plated on 6-well tissue culture polystyrene (TCPS) plates. Each well was filled with 2 mL of CO_2_ independent medium and cultured at 37 °C under atmospheric CO_2_ for 24 h. The cells were maintained in tissue culture flasks and cultured as monolayers until a density of 0.5 × 10^5^ cells/mL was achieved.

CRL-2314 cells were cultured in a tissue culture flask in RPMI 1640 medium with 2 mM L-glutamine adjusted to contain 1.5 g/L sodium bicarbonate, 4.5 g/L glucose, 10 mM HEPES, and 1.0 mM sodium pyruvate, 90% and fetal bovine serum, 10%.

**Figure 10:**
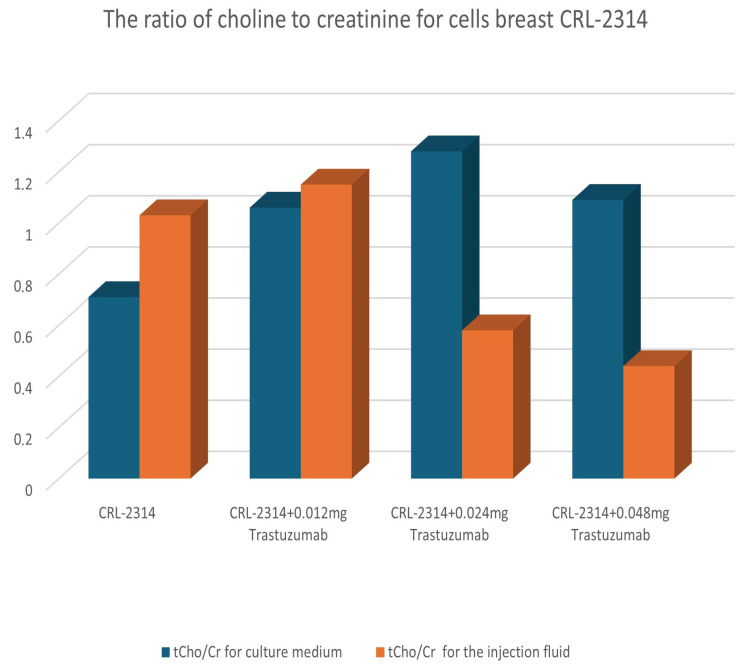
Comparison of treatment results for CRL-2314 cells cultured in injection fluid and culture medium.

3D cell cultures of MCF-7 and CRL-2314 cells: We used a Hollow Fiber Device to grow MCF-7 and CRL-2314 breast cancer cell lines in a controlled manner. MCF-7 cells and CRL-2314 cells (0.5 × 10^5^ cells/mL) were seeded in the Hollow Fiber Device. The bioreactor (Figure 10) was a closed loop system composed of porous hydrophilic hollow fibers with 0.1 µm size pores in a polysulfone tube. For this study, we used one fiber in each HFB cartridge. The polysulfone fiber was coated with 10 mL of collagen solution (1 mg collagen per 1 mL of phosphate-buffered saline (PBS) and 10 mL of fibronectin solution (10% in culture media). After the inoculation, the HFB device was perfused using a peristaltic pump. The flow of medium was adjusted at the rate of 5 mL/min and gradually increased to 14 mL/min. The pH was maintained between 6.8 and 7.0 in the extracapillary space throughout the duration of the experiments. The perfusion medium was changed weekly when the glucose level reached 2 g/L as measured with a glucometer.

In the experiment, we used the MCF-7 breast cancer cell line (American Type Culture Collection, Manassas, VA, USA) and the CRL-2314 breast cancer cell line (American Type Culture Collection, Manassas, VA, USA). Cells were cultured in standard conditions: temperature 37 °C, 5% CO_2_, and 95% humidity. EMEM medium (EBSS), 2 mM glutamine, 1% essential amino acids NEAA and 10% fetal bovine serum FBS/FCS were used to culture the MCF-7 cell line.

For the CRL-2314 cell line, the following culture medium was prepared based on an RPMI-1640 (Sigma-Aldrich, St. Louis, MO, USA), Fetal Bovine Serum (FBS) (Biochrom, Berlin, Germany), and the Penicillin antibiotic (Sigma-Aldrich, St. Louis, MO, USA). The procedure for preparing the final culture medium for CRL-2314 consisted of heating all of the components of the medium to a temperature of 37 °C, and then preparing the complete growth medium as follows: 3 mL of Fetal Bovine Serum and 300 µL of Penicilin antibiotic were added to 27 mL of RPMI-1640 medium. In the last step, the samples were centrifuged (5 min, 250× *g* in room temperature) in 1.5 mL Eppendorf tubes (Eppendorf, Hamburg, Germany) and MRS measurements were carried out.

Cell viability: The number of cells was determined using the Trypan blue exclusion method. Briefly, MCF-7 cells and HMEC cells are harvested from the HFB device, seeded in 6-well microplates, and exposed to 0.4% (*w*/*v*) Trypan blue dye solution. The cell numbers in the HFB device are also determined manually in a hemocytometer chamber. A commercially available, enzyme-linked immunosorbent assay kit (Sigma-Aldrich, St. Louis, MO, USA) is used for this purpose. Fresh medium is added to replace the used medium when the lactate concentration of the reservoir meets or exceeds 1000 lactate units/mL. When the number of the cells in the cultures reaches or exceeds 10^9^, the following tissue parameters relevant to cancer development are measured: water density, lactate, choline, glucose, and collagen content.

In the first step, the CRL-2314 breast cancer cells were tested. Then, a loading dose of Trastuzumab dissolved in the culture medium (i.e., 0.012 mg/mL) was added, and the cells were incubated for 24 h. The culture medium was changed sequentially, and the cells were incubated again for 24 h after which an increase in the number of cells was noticed. A double dose was then added and incubated again for 24 h.

In the second stage of the study, the same steps were repeated, only Trastuzumab was dissolved in water for injection. Two million cells were treated with Trastuzumab at 0.012 mg/mL for 24 h. Following incubation, the cells, as pellets, were collected by trypsinization and washed twice with a phosphate-buffered saline solution (PBS) before re-suspending in 1 mL PBS for further analysis. Finally, the cells were collected as pellets again by centrifugation at 1200 rpm for 10 min at room temperature. The same concentrations in water for injection were prepared to eliminate the effect of additional cell nutrition during the applied therapy. First, pure CRL-2314 cells were tested, then a loading dose was added to the culture and cells were incubated for 24 h (i.e., 0.012 mg/mL). The next step was 0.024 mg/mL and 0.048 mg/mL.

At each stage of the experiment, CRL-2314 cells were subjected to an abundance check. For cell counting, 20 µL of the cell suspension was taken and appropriately diluted in PBS, then the number of cells was automatically counted using Merck’s Muse Cell Analyzer count and viability kit. The results of the number and viability of CRL-2314 cells after the application of the Trastuzumab solution prepared in the medium are shown in Table 1. Cells were counted using a Muse Cell Analyzer (Merck, St. Louis, MO, USA; Millipore, Burlington, MA, USA). Cells were counted after applying the individual treatment steps after 24 h of incubation under standard conditions: 37 °C, 5% CO_2_, and 95% humidity.

Sample metabolite extraction. Two million cells per flask were used for each sample to avoid the effect of variable cell numbers. The cells were vortexed for 2 min to ensure the quantitative extraction of metabolites and then stored in ice for 1 h.

Visualization of cellular uptake of Trastuzumab: The MRS studies of the MCF-7 and CRL-2314 breast cancer cell lines using a clinical scanner with a 1.5 T field were performed. Before treatment, ^1^H MR images of MCF-7 and CRL-2314 cells were performed using a spin echo imaging sequence with TR = 1000 ms, TE = 40 ms, 0.5 mm slice thickness, a 2 cm × 2 cm field of view, 256 × 256 matrix size, and 10 signal averages. ^1^H MRS was acquired using a one-pulse sequence (flip angle 60°; repetition time (TR) = 800 ms; number of averages 2; echo time (TE) = 6 ms). 

Due to different chemical environments, the proton signals vary for Trastuzumab alone and Trastuzumab attached to the Her-2 receptor.

An attempt was made to use clinical MRI to monitor the response to Trastuzumab therapy in in vitro studies of breast cancer cell lines. Trastuzumab (150 mg) in the form of a powder for the preparation of a solution for infusion was obtained from “Roche Polska Sp. z.o.o.” (Warsaw, Poland). In addition to Trastuzumab, the 150 mg vial of Trastuzumab formulation consisted of L-histidine HCl, L-histidine, D-trehalose dihydrate, and polysorbate 20.

In order to accurately analyze the spectra of Trastuzumab, analyses of the spectra of the individual components were performed: D-trehalose dihydrate > 99% from Sigma Aldrich, L-histidine > 99% (TLC) from Sigma Aldrich, and the commercially available polysorbate 20.

Elaboration of MRS data acquisition parameters and processing. Data were processed using the program supplied with the MR scanner—SAGE7.7.1. This is a specialized package that allows the analysis of spectroscopic tests based on raw data from the MR scanner. In this system, many operations can be performed that affect the final appearance of the spectrum. For the purpose of analyzing the spectra included in this work, it was necessary to attenuate the water signal. As we know, this is a big problem in MRS spectroscopy, where the level of metabolites of interest can be many times lower than the level of the water signal—sometimes up to 10,000 times. This package enables water attenuation, but the attenuation band can be changed. The frequency at which the signal is attenuated is the frequency of the peak with the highest amplitude—in the case of MRS studies, it is most often the water signal. Although the level of water attenuation can be changed within wide limits, it should be remembered that the signal is digitally processed and its consequence is a significant reduction in signals located around the frequency of the main peak, i.e., approximately 4.7 ppm. In practical applications, the band between 4.5–5.2 ppm is strongly attenuated, which leads to a significant reduction or complete removal of some peaks in this range. Therefore, it is important that this tool be used as little as possible. For the research presented in this work, the attenuation band ranged from 10–40 Hz. The value of the LB parameter was within the range of 0.3–2 Hz and was selected for the best display of the spectrum. Increasing this parameter affects the appearance of the spectrum and smoothes it. An excessive LB value can cause some of the resonance signals to be merged, which means that the spectrum will have a lower resolution. MR systems are characterized by a lower homogeneity of the B0 magnetic field compared to NMR systems. The field is leveled before the test, but only until the optimal value is achieved, which does not guarantee the best field homogeneity in the area of interest. It was therefore necessary to fine-tune the field to ensure uniformity. This allowed us to obtain the best possible spectra of the tested objects. The use of a dedicated coil for research also improved the quality of the spectra. This is related to the size and shape of the examined object. The presentation of the MRS results itself, which is possible in several variants, took place in the “Power” window. Clinical systems are optimized for conducting research on the human body. The internal temperature of a human being is approximately 37 °C. In vitro sample testing involves recording the MRS signal at temperatures that are as much as 17 degrees Celsius lower. Omitting this factor may result in the incorrect assignment of spectra. Therefore, it was necessary to measure the temperature each time with a laboratory thermometer with a resolution of 0.1 degrees Celsius and to correct this temperature in the SAGE program, which took these changes into account. Due to the fact that electronic thermometers cannot be used in the MRI environment, it was necessary to use a mercury thermometer.

Coils: The MRS experiments were performed using the General Electric Healthcare (Milwaukee, Brookfield, WI, USA) Optima MR360 clinical magnetic resonance system with a field strength of 1.5 Tesla, software version SV23. This system was equipped with a series of coils; it is a device for clinical use that allows for the examination of all areas of the human body. This system; however, does not allow for the testing of such small objects as cell cultures. To enable data acquisition in this system, it was necessary to create a dedicated receiving coil. This design was developed for the study of small objects placed in Eppendorf tubes. Enameled copper wire with a diameter of 1.8 mm was used for matching the coil with the size and shape of the samples to obtain optimal conditions for RF data acquisition. This coil allows for obtaining both MR images as well as MRS for spectroscopic analysis and was connected to the MR system using the factory single-channel coil adapter.

**Figure 11:**
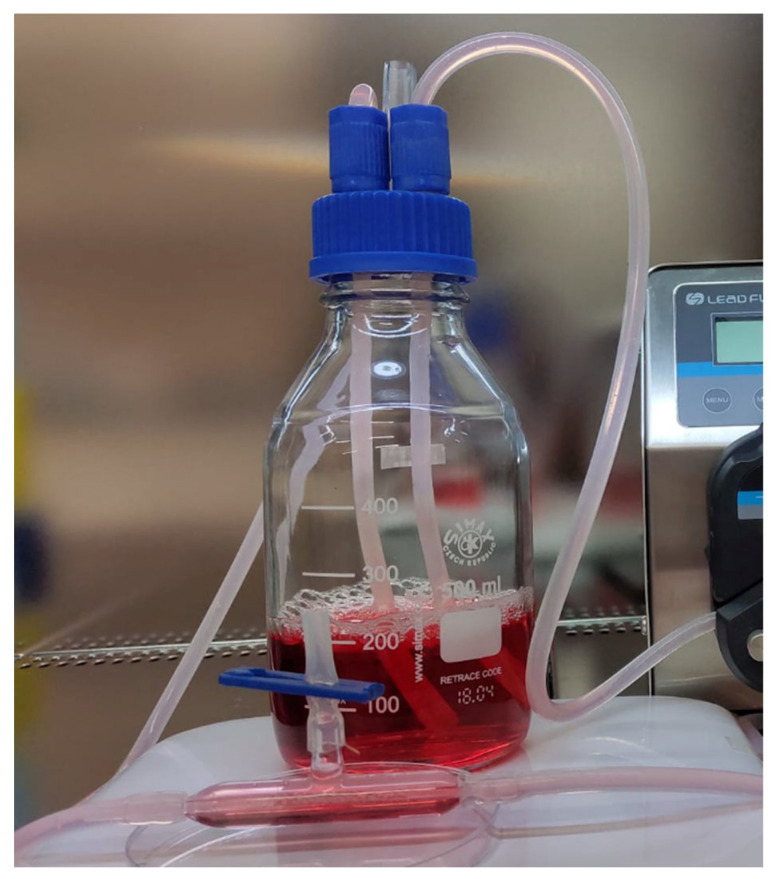
View of the bioreactor.

**Figure 12:**
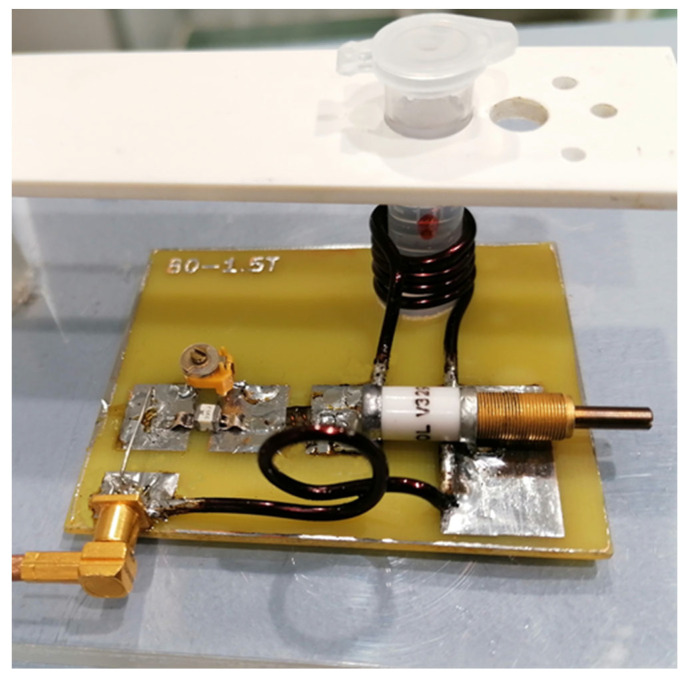
View of the receiving circuit during the test.

Figure 11 shows the receiving circuit and the main element of the receiving coil, a solenoid with a diameter of approximately 11 mm. After assembly, the receiving coil was tuned and tested for frequency response (Figure 12) with a RIGOL type: DSA815 spectrum analyzer. This device allows for the testing of the connected circuit in the 9 kHz to 1.5 GHz band.

**Figure 13:**
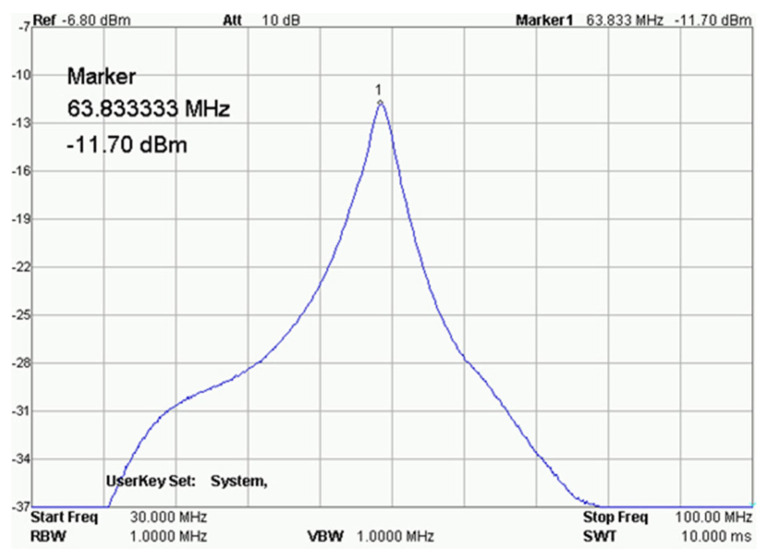
Result of the receiving coil analysis. RIGOL type: DSA815.

The prepared transmit–receive (RF) coil was constructed in-house (Figure 12). The following parameters were used for the SV sequence: voxel size 0.8 cm × 0.8 cm × 0.8 cm, minimum possible repetition time TR = 1149 ms and minimum echo time TE = 27 ms, and NEX = 8. The obtained results in the form of raw data files were analyzed using SAGE 7.7.1 software (Figure 13). (GE Medical Systems). In addition, in order to confirm the MRS spectra, experiments were performed using a 300 MHz NMR Fourier 300 with the TopSpin 3.1 system from Bruker.

## 5. Conclusions

The designed receiving coils allowed for conducting experiments with the cell lines in a satisfactory manner. These tests would not be possible using factory-delivered coils due to their parameters and the size of the test objects, whose volume did not exceed 1 mL. The MRS studies revealed an increase in the metabolite at 1.9 ppm, which indicates the induction of histone acetylation. Changes in histone acetylation play a very important role in both cell development and differentiation processes. The use of Trastuzumab therapy in breast cancer cells increases the levels of acetylated histones. MRS studies and spectra obtained from the 300 MHz NMR system are consistent with the specificity inherent in both systems.

MRS 1.5 T systems allow for conducting cell culture research to a limited but fully acceptable extent. Although they are not equal to NMR systems in terms of the resolution and quality of the spectra, they have an undeniable advantage over them. In MRS, there is no need to prepare cultures for testing in a destructive manner. This is due to the lower quality of the obtained spectra, but research on a given culture can be carried out many times, monitoring the progress of changes occurring in it. In summary, it should be stated that medium-field MR systems have great potential in the field of MRS research.

## Figures and Tables

**Table 1:** Identified MRS signals. All the experiments were performed in three replicates and each sample was analyzed three times.

Detected Cellular Composition	Untreated MCF-7	Treated MCF-7
Glutamine;	x	x
Creatine	x	x
Total Choline	x	x
Taurine	x	x
Glycine	x	x
PCho	x	-
GPC	x	x
Alanine	x	x
Pylosorbate 20	-	x
L-Histidine	-	x
D-Trehalose	-	x
Trastuzumab	-	x

x: present; -: absent.

**Table 2:** The number of viable CRL-2314 cells before and after Trastuzumab treatment.

Sample	Viable Cells/mL	Viability [%]	Total Cells/mL
CRL-2314	9.7 × 10^5^ ± 0.3 × 10^5^	93.1	1.04 × 10^6^ ± 0.03 × 10^6^
CRL-2314 + 0.012 mg Trastuzumab	1.43 × 10^6^ ± 0.05 × 10^6^	93.3	1.54 × 10^6^ ± 0.05 × 10^6^
CRL-2314 + 0.024 mg Trastuzumab	2.43 × 10^6^ ± 0.08 × 10^6^	89.6	2.72 × 10^6^ ± 0.08 × 10^6^
CRL-2314 + 0.048 mg Trastuzumab	2.1 × 10^6^ ± 0.07 × 10^6^	95.3	2.21 × 10^6^ ± 0.07 × 10^6^

**Table 3:** The results of the number of CRL-2314 cells before and after the application of the therapy with the use of the drug dissolved in the culture medium.

Sample	Viable Cells/mL	Viability [%]	Total Cells/mL
CRL-2314	1.08 × 10^7^ ± 0.03 × 10^7^	96.3	1.12 × 10^7^ ± 0.03 × 10^7^
CRL-2314 + 0.012 mg Trastuzumab	1.71 × 10^7^ ± 0.06 × 10^7^	98.9	1.73 × 10^7^ ± 0.03 × 10^7^
CRL-2314 + 0.024 mg Trastuzumab	1.07 × 10^7^ ± 0.03 × 10^7^	98.1	1.09 × 10^7^ ± 0.03 × 10^7^
CRL-2314 + 0.048 mg Trastuzumab	8.09 × 10^6^ ± 0.25 × 10^6^	98.7	8.2 × 10^6^ ± 0.25 × 10^6^
